# Comparison of drug-coated balloon and conventional balloon in autologous arteriovenous fistula stenosis

**DOI:** 10.1097/MD.0000000000042977

**Published:** 2025-06-27

**Authors:** Ningsu Kang, Yuxiang Qiu, Xi Wang, Pei Zhang, Jun Cui, Lu Zheng

**Affiliations:** a Department of Nephrology, Affiliated Nantong Hospital 3 of Nantong University, Nantong Third People’s Hospital, Nantong City, China.

**Keywords:** arteriovenous fistula, conventional balloon, drug-coated balloon, stenosis

## Abstract

This study compares the effects of drug-coated balloon (DCB) and conventional balloon (CB) in arteriovenous fistula (AVF) stenosis. We retrospectively analyzed the clinical data of 149 patients with AVF stenosis admitted to our hospital between June 2021 and October 2023 in this retrospective study. Among them, 73 and 76 patients were treated with CB (CB group) and DCB (DCB group), respectively. The treatment outcomes, peak flow velocity at the stenosis site, dialysis blood flow rate, vessel diameter, biochemical index levels, and incidence of complications were compared between the 2 groups. The success rate of surgery in the DCB group and the patency rate of AVF were higher than those in the CB group (97.37% vs 89.04%, 86.84% vs 71.23%, respectively; *P* < .05), and the length of hospital stay was longer in the DCB group than in the CB group (*P* < .05). After surgery, the peak flow velocity at the site of fistula stenosis in the DCB group was lower than that in the CB group, whereas the dialysis blood flow and vascular diameter were greater than those in the CB group (*P* < .05). After surgery, serum levels of angiotensin II, vascular endothelial growth factor-A, monocyte chemotactic protein-1, and ceramide were lower in the DCB group than in the CB group (*P* < .05). The incidence of complications in the DCB group was lower than that in the CB group (2.63% vs 10.96%; *P* < .05). Compared with CB, DCB has higher benefits in treating AVF stenosis.

## 1. Introduction

Hemodialysis is the mainstay therapy for patients with end-stage kidney disease.^[1]^ Arteriovenous fistula (AVF) is considered the preferred form of vascular access for patients requiring hemodialysis.^[1,2]^ However, during the use of AVF, factors such as calcification, puncture, and hemodynamic changes can easily cause fistula stenosis or occlusion, affecting the long-term patency of AVF and being detrimental to the prognosis of the disease.^[3,4]^ Therefore, effective treatment of AVF stenosis remains a research hotspot.

Ultrasound-assisted percutaneous transluminal angioplasty (PTA) is an important measure for the clinical management of AVF stenosis. PTA restores stenotic vascular access through balloon dilatation, which is influenced by balloon type, diameter, length, material, and inflation pressure. However, there is still a lack of a unified definition for the selection of balloons.^[5,6]^ Research has shown that conventional balloons may still lead to recurrent restenosis, whereas the drug components in the drug-coated balloon (DCB) group can inhibit endometrial hyperplasia, avoid restenosis, and thus improve patency.^[6–8]^

At present, there are few research results on the application of DCB in hemodialysis vascular access in China. To this end, we conducted a single-center retrospective study to compare the efficacy of DCB and conventional balloon (CB) in the treatment of autologous AVF stenosis. Explore the application effectiveness and safety of DCB and CB.

## 2. Materials and methods

### 2.1. Patients inclusion

In this retrospective study, a total of 149 patients with AVF stenosis admitted to our hospital between June 2021 and October 2023 were retrospectively selected. According to the different treatment methods of patients, they were divided into CB group (n = 73) and DCB group (n = 76).

### 2.2. Ethical approval

All procedures performed in this study involving human participants were in accordance with the ethical standards of the institutional and/or national research committee, and the 1964 Declaration of Helsinki and its later amendments or comparable ethical standards. Our study was approved by the Ethics Review Board of Affiliated Nantong Hospital 3 of Nantong University (No. NTSYL20240302, Date: March 6, 2024).

### 2.3. Inclusion criteria

(1) Patients met the diagnostic criteria for AVF stenosis;^[2]^ (2) Hemodialysis for ≥ 6 months; (3) No previous history of PTA; (4) The absolute value of the inner diameter of narrowed blood vessels was <2.5 mm or more than 50% compared to adjacent normal blood vessel stenosis; (5) CB or DCB treatment; (6) Age ≥ 18 years old; (7) The clinical data were complete.

### 2.4. Exclusion criteria

(1) Complex AVF (such as central venous stenosis, autologous complex AVF, etc); (2) acute thrombosis of AVF; (3) chronic occlusion of blood vessels, difficulty in passing through guide wires; (4) coagulation dysfunction patients; (5) individuals with vascular malformations; and (6) presence of infected individuals.

### 2.5. Method

Both groups underwent percutaneous endovascular angioplasty (PEA). The equipment selected is A Philips EPIQ-5 fully digital ultrasound imaging diagnostic instrument was used to explore the stenosis of AVF blood vessels and the intended dilation of blood vessels. The brachial artery was punctured using the Seldinger technique and was inserted into the vascular cavity. Stenosis of the AVF was identified using ultrasonography. To guide patients to lie flat and receive local anesthesia, ultrasound-assisted puncture is performed at a site 3 cm or more from the stenosis of the AVF anastomosis. The CB group adopted CB for expansion, and a dilator was inserted along the puncture needle to expand the skin and soft tissue. The sheath was inserted along the guide wire into the blood vessel, ensuring that residual stenosis at the opening of the vessel was ≤ 30%. The vessel-to-balloon diameter ratio was 1:1. Pressure was slowly applied during balloon dilation at the branch opening with a pressure of 760 mm Hg every 3 to 5 seconds. The maximum pressure was maintained for 60 seconds. The DCB group received treatment with paclitaxel drug release coronary balloon catheter; ultrasound-assisted puncture, insertion of guide wire, and insertion of puncture sheath. Subsequently, the guide wire was removed, the ultra-smooth guidewire was sent to the narrow area, the balloon pressure was implemented through a pressure pump, and the maximum pressure value was maintained for 60 seconds. Ultrasound-assisted monitoring of the dilation process, waiting for smooth blood circulation and disappearance of stenosis, removal of the balloon, super smooth guide wire, puncture sheath, and suture of the puncture site.

### 2.6. Evaluation indicators

(1) Treatment status, including surgical duration, surgical success rate, AVF patency rate, and length of hospital stay; surgical success: residual stenosis ≤ 30% detected by ultrasound imaging diagnostic instrument; AVF patency: blood flow rate during hemodialysis was ≥ 200 mL/min. (2) Peak flow velocity, dialysis blood flow rate, and vessel diameter at the site of AVF vascular stenosis were tested using ultrasound imaging diagnostic equipment. (3) Biochemical indicators, including levels of angiotensin II (Ang II), vascular endothelial growth factor-A (VEGF-A), monocyte chemotactic protein-1 (MCP-1), and ceramide (CER), extract 3 mL of blood sample, centrifuge, collect the supernatant, and measure the levels of the above indicators by enzyme-linked immunosorbent assay. (4) Complications: including bleeding, hematoma, hand-back swelling, and balloon/vascular rupture.

### 2.7. Statistical analysis

Data were analyzed using SPSS version 26.0 (IBM Corp, Armonk, NY). The measurement data were reported as mean ± standard deviation, and *t* test were used for comparison between the 2 groups. Categorical variables were reported as frequency and percentage, and comparison between the 2 groups were using chi-square or Fisher exact test. *P* < .05 was considered statistically significant.

## 3. Results

A total of 149 patients with AVF stenosis met the inclusion and exclusion criteria were included in this study. There were 73 patients of the CB group (40 male and 33 female) and 76 patients of the DCB group (39 male and 37 female). The age of the patients were between 38 and 81 (mean, 59.05 ± 10.76) years. The general data of sex, age, body mass index, hemodialysis time, duration of fistula application, length of stenosis, and primary disease were comparable between the 2 groups (*P* > .05) (Table 1).

**Table 1:** Comparison of patient characteristics between the 2 groups.

Characteristics	DCB group (n = 76)	CB group (n = 73)	*t*/*χ*^2^	*P*-value
Gender (male/female)	39/37	40/33	0.181	.681
Age (yr)	59.41 ± 10.44	58.68 ± 11.14	0.409	.683
BMI (kg/m^2^)	23.48 ± 2.74	22.98 ± 2.82	1.105	.271
Hemodialysis time (yr)	4.08 ± 1.73	4.25 ± 1.91	‐0.562	.575
Application duration of internal fistula (yr)	3.11 ± 1.65	3.29 ± 1.85	‐0.636	.526
Stenosis length (cm)	2.02 ± 0.73	2.09 ± 0.71	‐0.655	.514
Primary disease, n (%)				
Diabetes nephropathy	42 (55.26)	33 (45.20)	0.3.698	.296
Hypertensive nephropathy	21 (27.64)	30 (41.10)		
Glomerulonephritis	11 (14.47)	7 (9.59)		
Others	2 (2.63)	3 (4.11)		

Others = including polycystic kidney disease, obstructive nephropathy, and unknown cause of AVF stenosis.

BMI = body mass index, CB = conventional balloon, DCB = drug-coated balloon, *t* = *t* test, *χ*^2^ = chi-square test.

There was no significant difference in the duration of surgery between the 2 groups (*P* > .05). The success rate of surgery in the DCB group (97.37%) and the 6-month postoperative fistula patency rate (89.04%) were significantly higher than those in the CB group (86.84%, 71.23%), and the length of hospital stay was significantly shorter than that in the CB group (*P* < .05) (Table 2).

**Table 2:** Comparison of treatment outcomes between 2 groups.

Group	n	Surgical duration (h)	Surgical success rate [n (%)]	Six months postoperative fistula patency rate [n (%)]	Hospitalization duration (d)
DCB group	76	1.81 ± 0.57	74 (97.37)	66 (86.84)	3.28 ± 1.173
CB group	73	1.88 ± 0.57	65 (89.04)	52 (71.23)	3.96 ± 1.24
*χ*^2^/*t*		‐0.736	4.124	5.506	‐3.452
*P*-value		.463	.042	.019	.001

CB = conventional balloon, DCB = drug-coated balloon, *t* = *t* test, *χ*^2^ = chi-square test.

**Figure 1.**
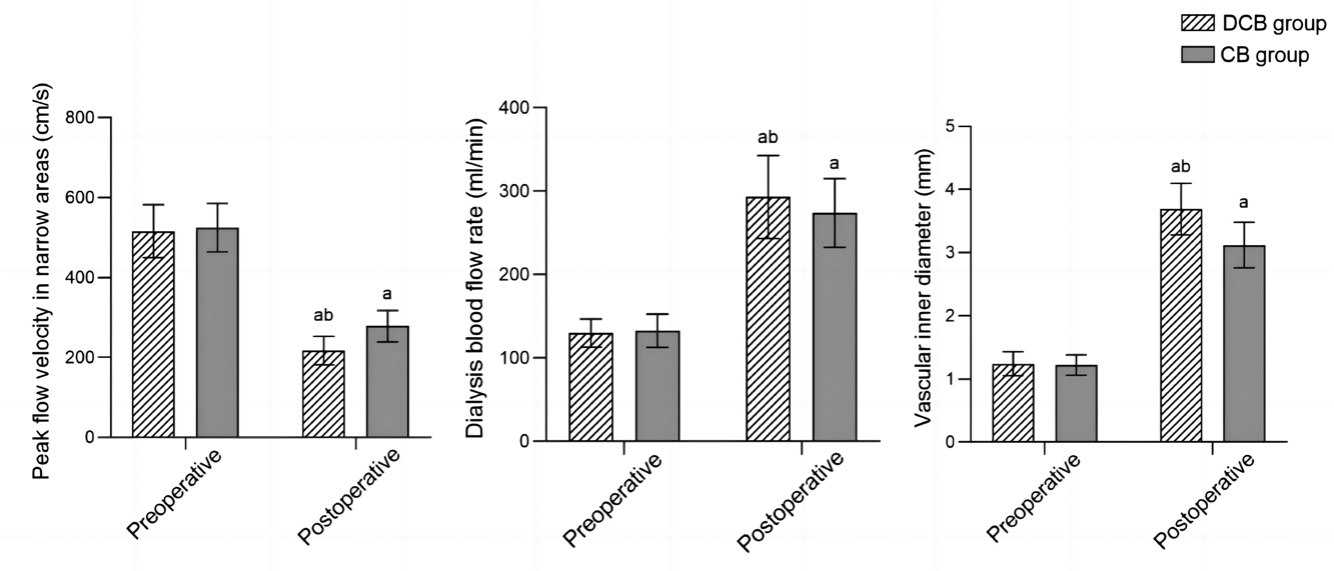
Comparison of peak flow velocity, dialysis blood flow rate, and vessel diameter at the site of stenosis in 2 groups of internal fistulas; ^a^*P* < .05, compared with the same group before surgery; ^b^*P* < .05, compared with the CB group after surgery. CB = conventional balloon, DCB = drug coated balloons.

Before surgery, there were no significant differences in peak flow velocity, dialysis blood flow rate, and vessel diameter between the 2 groups of AVF stenosis sites (*P* > .05). After surgery, the peak flow velocity at the site of stenosis in the 2 groups of AVF vessels decreased compared to before surgery, while the dialysis blood flow and vessel diameter were significantly higher than those before surgery. Moreover, the peak flow velocity at the site of fistula stenosis in the DCB group was significantly lower than that in the CB group, while the dialysis blood flow and vascular diameter were significantly higher than those in the CB group (*P* < .05) (Fig. 1).

**Figure 2.**
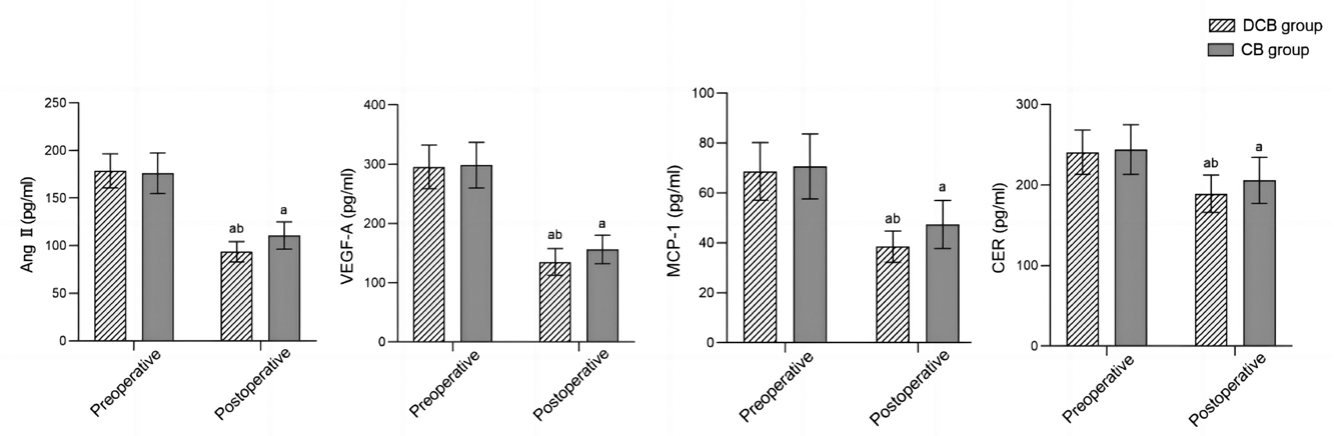
Comparison of biochemical indicators before and after surgery between 2 groups; ^a^*P* < .05, compared with the same group before surgery; ^b^*P* < .05, compared with the CB group after surgery. Ang II = angiotensin II, CB =conventional balloon, CER = ceramide, DCB = drug coated balloons, MCP-1 = monocyte chemotactic protein-1, VEGF-A = vascular endothelial growth factor-A.

Before surgery, there was no significant difference in serum Ang Ⅱ, VEGF-A, MCP-1, and CER levels between the 2 groups (*P* > .05). After surgery, the serum levels of Ang Ⅱ, VEGF-A, MCP-1, and CER in both groups were significantly reduced compared to before surgery, and the DCB group was significantly lower than the CB group (*P* < .05) (Fig. 2).

The incidence of complications in the DCB group (2.63%) was significantly lower than that in the CB group (10.96%) (*P* < .05) (Table 3).

**Table 3:** Comparison of incidence rates of complications between 2 groups.

Group	n	Oozing blood	Hematoma	Swelling of the back of the hand	Balloon/vascular rupture	Overall incidence rate
DCB group	76	0 (0.00)	1 (1.32)	1 (1.32)	0 (0.00)	2 (2.63)
CB group	73	2 (2.74)	3 (4.11)	2 (2.74)	1 (1.37)	8 (10.96)
*χ* ^2^						4.124
*P*-value						.042

CB = conventional balloon, DCB = drug-coated balloon, *χ*^2^ = chi-square test.

## 4. Discussion

Currently, the main intervention methods for AVF stenosis are PTA and vascular surgery. PTA has been recommended as the primary treatment for AVF stenosis by the clinical practice guidelines of Kidney Disease Outcome Quality Initiative and the European Society for Vascular Surgery.^[9,10]^ PTA uses balloons to dilate the stenotic area of the blood vessel. CB is usually made of latex or silicone, and its bursting pressure is generally (12–14) × 10^5^ Pa. The balloon should not be inflated for too long. It usually lasts for 1 to 3 minutes each time and is repeated 1 to 2 times until the balloon pressure mark disappears on ultrasound. Although CB angioplasty has a high success rate, it is prone to acute elastic recoil of the vessel, restenosis, and with limited long-term patency.^[11,12]^ At present, a new type of DCB is being applied in blood dialysis vascular pathways. The most commonly used drug ingredient is paclitaxel, because it has high lipophilicity and can be quickly absorbed by the vascular wall and retained in endothelial cells after short contact. Various complex mechanisms mediate cell apoptosis, inhibit neointimal proliferation, prolong patency time, and delay the occurrence of restenosis.^[13,14]^

The results of this study indicate that compared to CB, the DCB group can more effectively increase dialysis blood flow and vascular diameter, improve surgical success and AVF patency rates, and reduce the risk of complications, promoting early recovery and hospital stay. The results of this study are consistent with the previous studies that comparing the effect of CB and DCB in treating AVF stenosis.^[15–17]^ It was reported that using paclitaxel layer balloon angioplasty for the treatment of recurrent AVF stenosis can improve the success rate of surgery and improve the patency of the fistula. This finding is consistent with the results of the present study.^[14]^ The DCB uses balloons as mediators to transport antiproliferative drugs to the stenosis site and evenly release them, which can effectively expand the diameter of the stenosis and increase dialysis blood flow.^[13]^ Paclitaxel can penetrate the blood vessel wall through a balloon. It is hydrophobic, can concentrate on the intima of blood vessels, has significant antiproliferative activity, and can effectively inhibit the proliferation of blood vessel intima, with a long duration of efficacy.^[13,18]^ DCB drugs can be effectively released in a short period of time, and after balloon removal, there is no foreign body residue in the target vessel. This not only inhibits smooth muscle proliferation but also reduces endothelialization delay and restenosis risk.^[16–18]^

However, some studies have not found that DCB is more effective than CB in treating AVF stenoses. Narayan et al^[19]^ conducted a multicenter randomized controlled trial on paclitaxel-coated balloons in AVF and confirmed that they do not have significant benefits for AVF. The research results of Cemal et al^[20]^ indicated that both CB and DCB groups have high application value, and there was no significant difference in the target lesion patency rate between the 2 groups at 6 months after surgery, which was 81.3% versus 93.1%. We question our views on the benefits that DCB can provide to patients with hemodialysis pathways based on the above research findings. It is still necessary to conduct extensive research in the future to explore the effectiveness of DCB in treating AVF stenotic lesions.

Research has shown that Ang II can bind to Ang II-1 receptors, stimulate vascular intimal proliferation and endothelial cell apoptosis, exacerbate vascular damage, and cause stenosis of AVF^[21]^; VEGF-A increases the level of plasma enzyme activating factor, accelerates extracellular protein hydrolysis, and accelerates the formation of new capillaries^[22]^; Elevated MCP-1 expression can accelerate smooth muscle cell migration and proliferation, thereby increasing the risk of AVF stenosis.^[23]^ Related studies have found that the DCB group can more effectively reduce the levels of Ang II, VEGF-A, and MCP-1 in patients with AVF stenosis.^[24,25]^ In addition, CER is an important lipid second messenger for endothelial cells and vascular smooth muscle cells, which can regulate the migration, proliferation, and phenotype transformation of endothelial cells, smooth muscle cells, and outer membrane myofibroblasts, increase the extracellular matrix, cause narrowing of the lumen, and result in AVF failure.^[26]^ This study found that the serum levels of Ang II, VEGF-A, MCP-1, and CER decreased in both groups after surgery compared with before surgery, and the DCB group was lower than the CB group. This indicates that the DCB group is superior to the CB group in regulating Ang II, VEGF-A, MCP-1, and CER levels, but its underlying mechanism needs to be further studied.

### 4.1. Limitations of the study

First, this was a single-center retrospective study with a small sample size of 149 cases, which may limit its generalizability of the findings to broader populations. Second, due to the small number of cases, further subgroup analysis was not conducted on the patient’s sex, age, dialysis age, primary disease, and the location of stenosis, which may have affected the patency of the internal fistula. Third, tracking long-term patient outcomes after treatment would be critical to determine the true efficacy and durability of DCB in preventing restenosis. The long-term effect of DCB on patient outcomes including the functionality of arteriovenous fistulas over time and quality of life were not analyzed in this study, nor was re-intervention rate. Finally, artificial vascular AVF and central venous catheterization related vascular pathways were not included in the study to explore the efficacy of the DCB group in treating stenosis of these vascular pathways. The study was conducted in a single center in China, and these results cannot be generalizable to other countries with different practices, population and ethnicity backgrounds.

## 5. Conclusion

Compared with CB, DCB has a higher benefit in treating AVF stenosis. In addition, future studies comparing DCB with other advanced or emerging treatment modalities would better clarify the clinical role of DCB.

## Author contributions

**Conceptualization:** Ningsu Kang, Lu Zheng.

**Data curation:** Ningsu Kang, Yuxiang Qiu, Lu Zheng.

**Formal analysis:** Ningsu Kang, Lu Zheng.

**Investigation:** Ningsu Kang, Yuxiang Qiu, Jun Cui.

**Methodology:** Xi Wang.

**Project administration:** Xi Wang.

**Resources:** Pei Zhang.

**Software:** Pei Zhang.

**Validation:** Jun Cui.

**Visualization:** Jun Cui.

**Writing – original draft:** Ningsu Kang, Lu Zheng.

**Writing – review & editing:** Lu Zheng.
